# Autoclaving is at least as effective as gamma irradiation for biotic clearing and intentional microbial recolonization of soil

**DOI:** 10.1128/msphere.00476-24

**Published:** 2024-07-09

**Authors:** William L. King, Emily M. Grandinette, Olivia Trase, M. Laura Rolon, Howard M. Salis, Harlow Wood, Terrence H. Bell

**Affiliations:** 1 Department of Plant Pathology and Environmental Microbiology, The Pennsylvania State University, University Park, Pennsylvania, USA; 2 School of Integrative Plant Science, Cornell University, Ithaca, New York, USA; 3 School of Biological Sciences, University of Southampton, Southampton, United Kingdom; 4 Department of Entomology, The Pennsylvania State University, University Park, Pennsylvania, USA; 5 Intercollege Graduate Degree Program in Ecology, The Pennsylvania State University, University Park, Pennsylvania, USA; 6 Department of Food Science, The Pennsylvania State University, University Park, Pennsylvania, USA; 7 Department of Agricultural and Biological Engineering, The Pennsylvania State University, University Park, Pennsylvania, USA; 8 Department of Physical & Environmental Sciences, University of Toronto Scarborough, Toronto, Ontario, Canada; University of Wisconsin-Madison, Madison, Wisconsin, USA

**Keywords:** autoclaving, microbial recolonization, soil sterilization, gamma-irradiation, biotic-constraints, soil microcosm

## Abstract

Sterilization is commonly used to remove or reduce the biotic constraints of a soil to allow recolonization by soil-dwelling organisms, with autoclaving and gamma irradiation being the most frequently used approaches. Many studies have characterized sterilization impacts on soil physicochemical properties, with gamma irradiation often described as the preferred approach, despite the lower cost and higher scalability of autoclaving. However, few studies have compared how sterilization techniques impact soil recolonization by microorganisms. Here, we compared how two sterilization approaches (autoclaving; gamma irradiation) and soil washing impacted microbial recolonization of soil from a diverse soil inoculum. Sterilization method had little impact on microbial alpha diversity across recolonized soils. For sterile soil regrowth microcosms, species richness and diversity were significantly reduced by autoclaving relative to gamma irradiation, particularly for fungi. There was no impact of sterilization method on bacterial composition in recolonized soils and minimal impact on fungal composition (*P* = 0.05). Washing soils had a greater impact on microbial composition than sterilization method, and sterile soil regrowth had negligible impacts on microbial recolonization. These data suggest that sterilization method has no clear impact on microbial recolonization, at least across the soils tested, indicating that soil autoclaving is an appropriate and economical approach for biotically clearing soils.

**IMPORTANCE:**

Sterilized soils represent soil-like environments that act as a medium to study microbial colonization dynamics in more “natural” settings relative to artificial culturing environments. Soil sterilization is often carried out by gamma irradiation or autoclaving, which both alter soil properties, but gamma irradiation is thought to be the gentler technique. Gamma irradiation can be cost prohibitive and does not scale well for larger experiments. We sought to examine how soil sterilization technique can impact microbial colonization, and additionally looked at the impact of soil washing which is believed to remove soil toxins that inhibit soil recolonization. We found that both gamma-irradiated and autoclaved soils showed similar colonization patterns when reintroducing microorganisms. Soil washing, relative to sterilization technique, had a greater impact on which microorganisms were able to recolonize the soil. When allowing sterilized soils to regrow (i.e., persisting microorganisms), gamma irradiation performed worse, suggesting that gamma irradiation does not biotically clear soils as well as autoclaving. These data suggest that both sterilization techniques are comparable, and that autoclaving may be more effective at biotically clearing soil.

## INTRODUCTION

Traditional culture-based approaches have a long history in microbiology and remain essential to microbiological research (1). Such approaches are ideal for certain research questions (e.g., physiological probing of easily captured species), but are limited in their ability to model complex microbiomes under realistic environmental conditions. In addition, most microbes remain difficult to cultivate, restricting in-culture studies to easily isolated microbes, which may be poor analogs for many of the important microbial players in complex environments, like soil (2–5).

To circumvent these limitations, soils are often “sterilized” to provide biotically cleared environments that can be reinoculated with specific microbes or intact microbiomes (6–11). This type of approach is controllable and reproducible; for instance, we can see strong differentiation of introduced microbiomes (6, 12–14). It is also a more realistic model of an in-field soil compared to culture media, both structurally and chemically. In fact, widespread agricultural practices use biotic clearing of soil to eliminate pathogens, so in-lab “sterilization” of soil is an excellent analog for such cases (12, 15, 16). However, the best approach for biotic clearing of soil has been debated for decades, since any sterilization method alters soil characteristics to some degree (17). As a result, biotically cleared soils should not be treated as perfect replicates of in-field soils, but as soil-like environments that are similar to, but not equivalent to, the pre-sterilized soil. Numerous approaches, including airdrying, oven drying, addition of chemicals or antibiotics, fumigation, microwaving, gamma irradiation, and autoclaving have been repeatedly tested as methods that can maximize sterilization while minimizing impacts on soil physicochemical characteristics (18).

Two common soil sterilization procedures are gamma irradiation and autoclaving (for long periods of time and/or multiple times over several days). Gamma irradiation requires highly specialized facilities and equipment, but has been described by some as a “gold standard,” as it is thought to cause fewer substantial changes to some soils during sterilization (17, 19, 20). Nonetheless, gamma irradiation is not well borne out across a wide range of soil types (21) and may not biotically clear soils better than autoclaving (19). Similar to other sterilization methods, gamma irradiation alters the physical and chemical properties of soil, including the release of highly reactive free radicals and changes to the structure of organic matter, among others (17, 22). Although less irradiation time and intensity can reduce the impact on soil properties, it may also be less effective in removing soil organisms, thus defeating its intended purpose. Autoclaving also alters a variety of soil physicochemical properties (17), but offers benefits that cannot be achieved with irradiation, such as ease of access and low financial burden, facilitating larger-scale soil processing to run experiments at scale (6, 7, 12–14, 17, 23, 24). Autoclaving closely mirrors large-scale soil management practices, such as soil steaming, which are used in agricultural settings to remove pathogens from the soil. Although numerous studies have investigated how each sterilization approach impacts soil structure and chemistry (17, 18), it is essential to understand the relative impacts of sterilization approaches on the growth of introduced microorganisms.

In this study, we aimed to determine the impacts of common sterilization approaches on the potential of soil microbiomes to colonize, establish, and persist within biotically cleared soils. In addition to the two most common approaches for soil sterilization (i.e., gamma irradiation and autoclaving), we included a washing step, as it has been suggested this could reduce the impact of released compounds or spores remaining in the soil after sterilization (25–32). Microbiomes from the pre-sterilized versions of two soils were introduced to assess the impact each sterilization technique had on the ability of microbes to colonize and persist within the biotically cleared soil environments. Based on prior in-lab observations, we hypothesized that (i) soil type would have a greater impact on resulting microbial composition than sterilization method, and that (ii) autoclaved soils would have less diverse microbial “contaminants” (regrowth), yielding more reproducible microbial composition relative to gamma-irradiated soil samples.

## MATERIALS AND METHODS

### Soil collection and substrate conditions

Two physicochemically distinct soils within a standard agricultural pH range were collected from The Pennsylvania State University Russell E. Larson Agricultural Research Center at Rock Springs (Table S1). Soil 1 was collected from an unmanaged, forested area, while Soil 2 was collected from a plot previously used for field trials of agricultural crops. Debris was cleared from the ground and a shovel was used to collect soil from the top 20 cm. Samples were sieved to 2 mm using a standard brass sieve and mixed 2:1 (vol/vol) with Quikrete All-Purpose Silica Sand to improve aeration and drainage. Between each stage (i.e., collection, sieving, sand addition, sterilization, and microcosm construction), soils were stored in sealed bags at 2°C. A fraction of each soil was left unmixed for use as negative controls.

### Autoclave-based sterilization

A portion of each prepared substrate was placed into standard biohazard bags (double-bagged) that are permissive to autoclaving and a soil depth of 2–3 cm. Each soil was autoclaved for 45 min at 121°C and 20 psi, with this process repeated three times total, including 24 h of room temperature incubation between cycles. This room temperature incubation is intended to allow spores that survived the first round of autoclaving an opportunity to germinate and become more vulnerable to a second round to improve biotic clearing. Autoclaving soils is a common technique that is generally performed for 2–3 cycles, with 24–48 h of incubation between each cycle, and allows for large-scale processing of soil (e.g., 6, 10, 11, 14, 17, 23–25, 33–35).

### Gamma irradiation-based sterilization

A portion of each prepared substrate was sent to The Pennsylvania State University’s Radiation Science and Engineering Center’s Gamma Irradiation Facility. Samples were double-bagged in polypropylene and irradiated at a dose of 50 kGy (17, 18, 36, 37), using Co^60^ neutron sources in a Gammacell 220 dry cell irradiation chamber. Samples were stored at 4°C while queued at the facility and stored at 2°C upon return (~10 days).

### Soil saturation test and wash

Portions of the sterilized substrate samples underwent a wash procedure, as it has been suggested that washing soil may eliminate compounds released during the sterilization process (25–30). In order to estimate the saturation point of each substrate, we used a modified version of the approach used in O. Priha and A. Smolander (38). We placed 5 g of unsterilized soil, 5 g of autoclaved substrate, and 5 g of irradiated substrate for each of the two soils onto separate layers of thin nylon. Nylon was used to allow drainage while retaining the bulk of the soil sample. Water was poured over the soil until the physical structure appeared to begin breaking down (max. added = 5 mL). Samples sat for 1 h to facilitate drainage. To estimate the saturation point, any excess water not added from the first pour step was combined with the drain through for each sample. The amount of water retained by the soil was determined to be the measured volume of the leftover and drained water subtracted from the initial 5 mL added.

Once this point was determined, autoclave-sterilized DI water (20 min at 121°C and 20 psi) was used to wash the soils. For each sterilized substrate, portions were spread over the top of a standard 250 µm brass sieve. Water was poured over the substrate (1:1 vol/vol ratio) to exceed the determined saturation points for each substrate, so that water would drain through the soil rather than being completely absorbed. Substrates were drained to remove excess water for 45 min. Sieves were wiped and ethanol-sterilized between each sample to avoid contamination. Once washed, each substrate was divided into sterile containers with loose fitting lids and stored at room temperature for 72 h to continue removing excess moisture through evaporation.

### Microcosm assembly and inoculation

For all microcosms, ~25 g of each type of soil or substrate was added to a small Petri dish (~50 mm × 10 mm). For recolonization (henceforth known as the natural recolonization method), small portions of unsterilized soil (~2 g) were placed in the center of small, ethanol-sterilized nylon squares. The nylon was gathered around the soil, pinched, and held closed with 24-gauge beading wire to create inoculation sachets. Excess nylon was cut from the top to ensure Petri dishes could still close with the sachets inside. To establish these microcosms, sachets were placed several millimeters in from the edge of a small Petri dish. Each substrate was added to surround the sachets on all sides, with the wire closure remaining visible above the surface of the surrounding soil. The soil inside the sachets had the same origin as the surrounding soil (i.e., if Soil 2 was placed inside the sachet, Soil 2 was also used to fill the microcosm). The sachets facilitate direct soil-to-soil contact (8), allowing microorganisms present in the unsterilized soil to pass into the surrounding sterilized soil, while minimizing the transfer of large soil particles.

Microcosms containing soil which was not washed prior to construction were saturated using autoclave-sterilized water. Volumes were chosen based on the previously described saturation estimates. No additional water was added to washed soils, as they were saturated during the washing procedure. Some microcosms were constructed with sterile soil only (no inoculum introduced) to allow for potential microbial regrowth in the absence of microbial inoculation. The microcosms were then wrapped with a thin layer of parafilm to prevent desiccation and incubated in a growth chamber for 8 weeks at 20°C and 85% relative humidity. Microcosms were randomly scattered across a single shelf within the growth chamber to minimize location bias.

### Microcosm sampling and vitality testing

After the 8-week incubation, microcosms were removed from the growth chamber and aseptically sampled. For each microcosm, two 1.5 mL Eppendorf tubes were filled with the soil farthest away from the marked inoculation point and stored at −80°C for future analysis.

A third Eppendorf tube was partially filled for one sample in each treatment; this sample was mixed 1:1 with sterile water, diluted to 10^−3^, and 25 µL was plated onto Tryptic Soy Agar. Plates were incubated at room temperature for 48 h. This allowed a preliminary visualization of whether microbes had survived the incubation. Once the viability of the samples was confirmed, samples were processed for amplicon sequencing.

### DNA extraction and amplicon sequencing

DNA was extracted from ~300 mg of each harvested soil sample using NucleoSpin 96 Soil kits (Machery-Nagel, Germany). Microbial composition was characterized within the 16S rRNA gene for bacteria (515F and 806R; V4 region) and internal transcribed spacer (ITS) region for fungi (ITS1F and 58A2R) using amplicon sequencing. The PCR ingredients for both reactions were as follows: 12 µL of 5Prime HotMasterMix, 1.5 µL of each primer (10 µM), 1.5 µL template DNA, and 13.5 µL molecular grade water for a final PCR volume of 30 µL. Bacterial 16S rRNA gene PCR cycling conditions were as follows: 3 min at 94°C, 25 cycles of 45 s at 94°C, 60 s at 50°C, and 90 s at 72°C, and a final elongation step of 10 min at 72°C. Fungal ITS PCR cycling conditions were as follows: 3 min at 94°C, 35 cycles of 20 s at 94°C, 30 s at 45°C, and 45 s at 72°C, and a final elongation step of 5 min at 72°C. The resulting amplicons were cleaned using Mag-Bind TotalPure NGS magnetic beads (Omega Bio-Tek; catalog: M1378-01), and cleaned amplicons were indexed, normalized, concentrated, and purified. Sequencing was performed at the Cornell University Biotechnology Resource Center Genomics Facility on an Illumina MiSeq using the 2 × 250 cycle v.2 kit. Raw reads are available in the NCBI Sequence Read Archive under BioProject number PRJNA727754.

### Sequence analysis

Raw demultiplexed 16S rRNA gene and ITS region data were processed using the Quantitative Insights into Microbial Ecology (QIIME 2 version 2020.11) pipeline (39). Briefly, paired-ended 16S rRNA gene and ITS region DNA sequences were imported and trimmed, and denoised using DADA2 which also removes chimeras (40). The classify-sklearn qiime feature classifier was used to assign taxonomy against the Silva v.132 (41) or UNITE v.04.02.2020 (v.8.2) database (42) at the single nucleotide threshold (zero-radius OTUs). The data set was further cleaned by removing sequences identified as chloroplasts or mitochondria. The cleaned 16S rRNA gene and ITS data were then rarefied at 5,732 and 2,430 sequences per sample, respectively.

### Statistical analysis

Statistical comparisons were performed in the R statistical environment (43) version 4.2.1. To identify differences in alpha diversity metrics (i.e., Chao1 species richness and Shannon species diversity) for the natural recolonization treatment, we used analysis of variance followed by a *post hoc* Tukey HSD (honestly significant difference) test from the stats package (version 4.2.1). Homogeneity of variance and normality of residuals were tested using the Levene’s and Shapiro-Wilk’s tests in the car (44) (version 3.1–2) and stats packages, respectively, and data were transformed when needed. A Kruskal-Wallis test was used to compare sterilization method for the soil regrowth soils. Bacterial and fungal compositional matrices were imported into the phyloseq R package (45) (version 1.4). We chose rarefaction and proportional transformation (i.e., relative abundance), as these produce the most accurate representations of community-level patterns and account for uneven sequencing depth (46, 47). Comparisons of microbial composition at the amplicon sequence variant (ASV) level were performed with a principal coordinates analysis (PCoA) ordination with a Bray-Curtis dissimilarity index and with a permutational multivariate ANOVA (PERMANOVA; Adonis2) from the vegan package (version 2.6-4) with 999 permutations (48). To examine shifts in taxa according to sterilization and wash treatment, we performed a SIMPER (similarity percentage) analysis from the vegan package on data summarized at the phylum and class levels for bacteria and fungi, respectively. To determine the degree of dissimilarity between sterile soil regrowth and microbial recolonization, we calculated Bray-Curtis dissimilarity values of recolonized soil relative to soil regrowth, which were compared using a Kruskal-Wallis test from the stats package and a *post hoc* Dunn test from the FSA package (49) (version 0.9.4).

### Assessment of microbial regrowth in the absence of inoculation across autoclave conditions

As we were limited in the number of soils and sample treatment conditions that we could test here, we performed a limited-scope follow-on study, in which we solely assessed the extent of microbial regrowth post-autoclaving in the absence of any microbial reinoculation. We tested regrowth across seven distinct soils collected from Koffler Scientific Reserve at Jokers Hill (King City, ON, Canada) and the Campus Farm at the University of Toronto–Scarborough, as well as six modified treatment conditions in a single soil. Standard conditions were three autoclave cycles, with soil kept to 1.5” depth, and field-level moisture retained, with the following modifications for treatments: (i) pre-drying of soil; (ii) reduction from three autoclave cycles to one; (iii) reduction from 1.5” to 0.5” depth; (iv) increase from 1.5” to 3” depth; (v) 1:1 mixture of soil:peat (vol:vol); and (vi) 1:1 mixture of soil:perlite. After 7 weeks of incubation in closed microcosms, DNA was extracted and quantified with a Qubit 4.0 fluorometer (Invitrogen, Carlsbad, CA, USA) across two separately incubated microcosms for each condition.

## RESULTS AND DISCUSSION

### More taxa were detected in gamma-irradiated soil regrowth microcosms

We compared autoclaving and gamma irradiation to determine whether sterilization method had a significant impact on soil recolonization by microorganisms or microbial regrowth. For the natural recolonization method, we generally did not detect differences between sterilization method and wash treatment for species richness and species diversity, except for fungal alpha diversity in Soil 1 (Tables S2 and S3) which was primarily driven by poor fungal recolonization (low alpha diversity values) in the washed autoclaved soils. These data indicate that sterilization method did not substantially impact alpha diversity in recolonized soils.

**Fig 1:**
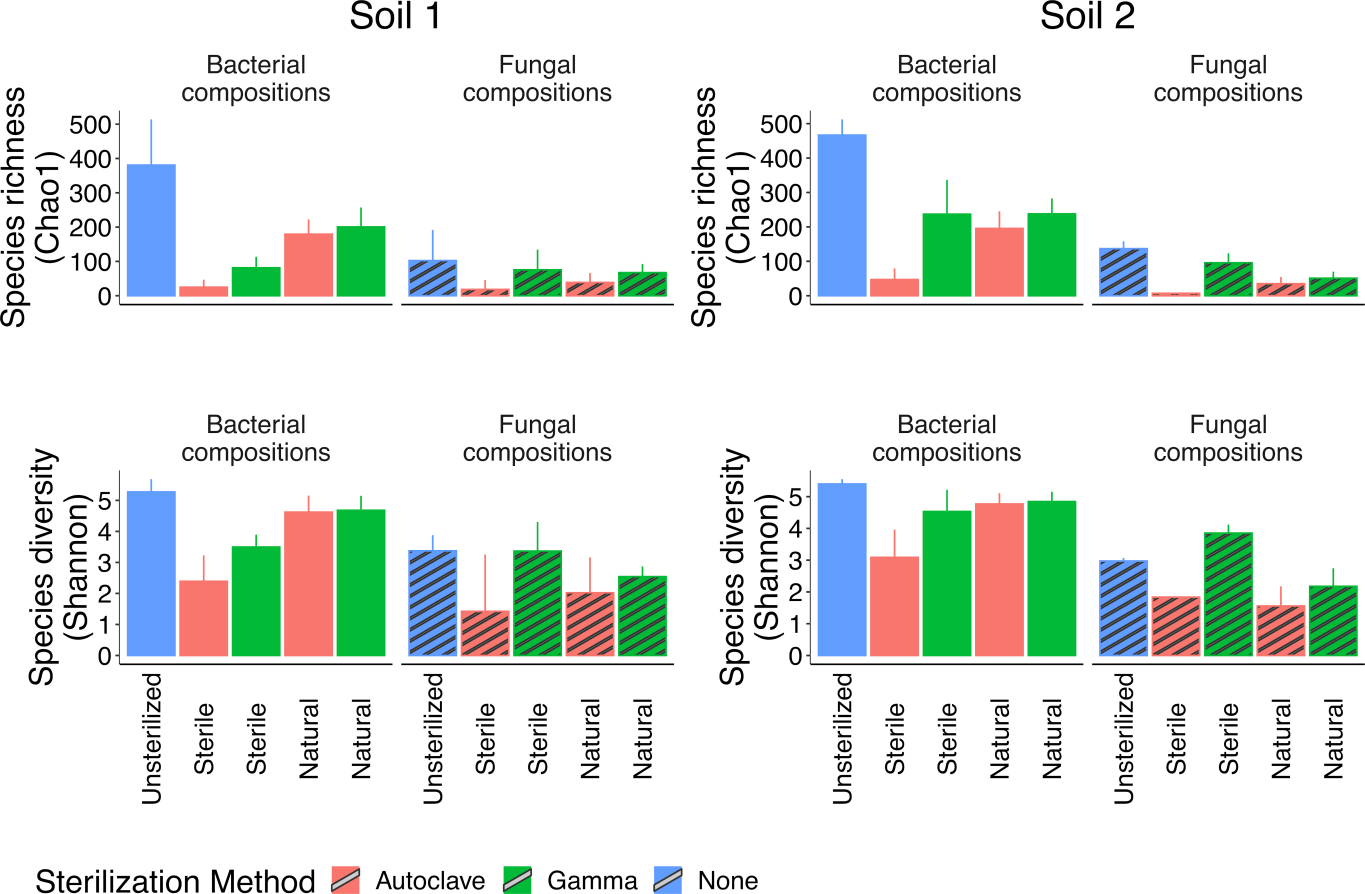
Alpha diversity measures for bacteria and fungi. “Unsterilized” is untreated soil; “Sterile” is sterile soil regrowth control; “Natural” is natural recolonization of biotically cleared soils from the unsterilized version of the same soil.

In a number of prior studies, we have seen that autoclaved soil had no detectable DNA or amplifiable PCR product shortly after sterilization and even after a period of incubation [e.g., L. M. Kaminsky et al. (12)]. Other studies have also shown a lack of viable organisms following both sterilization approaches [e.g., A. E. Berns et al. (17)]. However, we have noted that the diversity and rate of microbial regrowth in sterilized soils differs by soil type, which is an area of interest for future studies. In our sterile regrowth controls, there was significantly greater bacterial species richness (Soil 1: H = 4, *P* = 0.04; Soil 2: H = 5, *P* = 0.02), and bacterial diversity in Soil 2 (H = 5, *P* = 0.02), regrowth in the gamma-irradiated soils relative to the autoclaved soils (Table S2). For fungal composition, we detected no significant differences for either species richness or species diversity in either soil. However, autoclaving had a particularly strong impact on the fungal soil regrowth controls (Fig. 1) which may explain the lack of significance. In Soil 1, we observed two replicates with Chao1 values of 2 and 3 and an outlier replicate at 50. In Soil 2, only one replicate made it through the sequence analysis, as two replicates were removed during cleaning (only three to eight input reads) and an additional replicate was removed during rarefaction (only 280 cleaned sequences). As a consideration, we did not account for differences in total DNA and our observations are based on the relative abundance of those samples that could amplify. There was 17× and 7× greater species richness in gamma-irradiated regrowth soils when compared to autoclaved regrowth for bacteria and fungi, respectively (Fig. 1). In support of our data, autoclaving has been shown to have a stronger negative impact on bacterial counts and CO_2_ production (19) and microbial cell lysis (17) relative to gamma irradiation. In this study, we used a gamma irradiation dose of 50 kGy. The efficacy of gamma irradiation can be impacted by soil properties, such as moisture content and organic matter content (18). Fungal composition is sensitive to gamma irradiation and can be impacted with doses of 0.01 kGy, with 10 and 50 kGy doses optimal to completely clear all viable fungi and spores in low and high organic matter soils, respectively (18). Furthermore, doses of 20–25 kGy have been suggested as sufficient to clear bacteria (18, 20). While our chosen dose should have adequately eliminated bacteria and fungi from our soils, in both soils, we observed trends toward substantially higher alpha diversity in the gamma-irradiated soil regrowth relative to the autoclaved soil regrowth control.

### Wash treatment had a greater impact than sterilization method on soil recolonization

**Fig 2:**
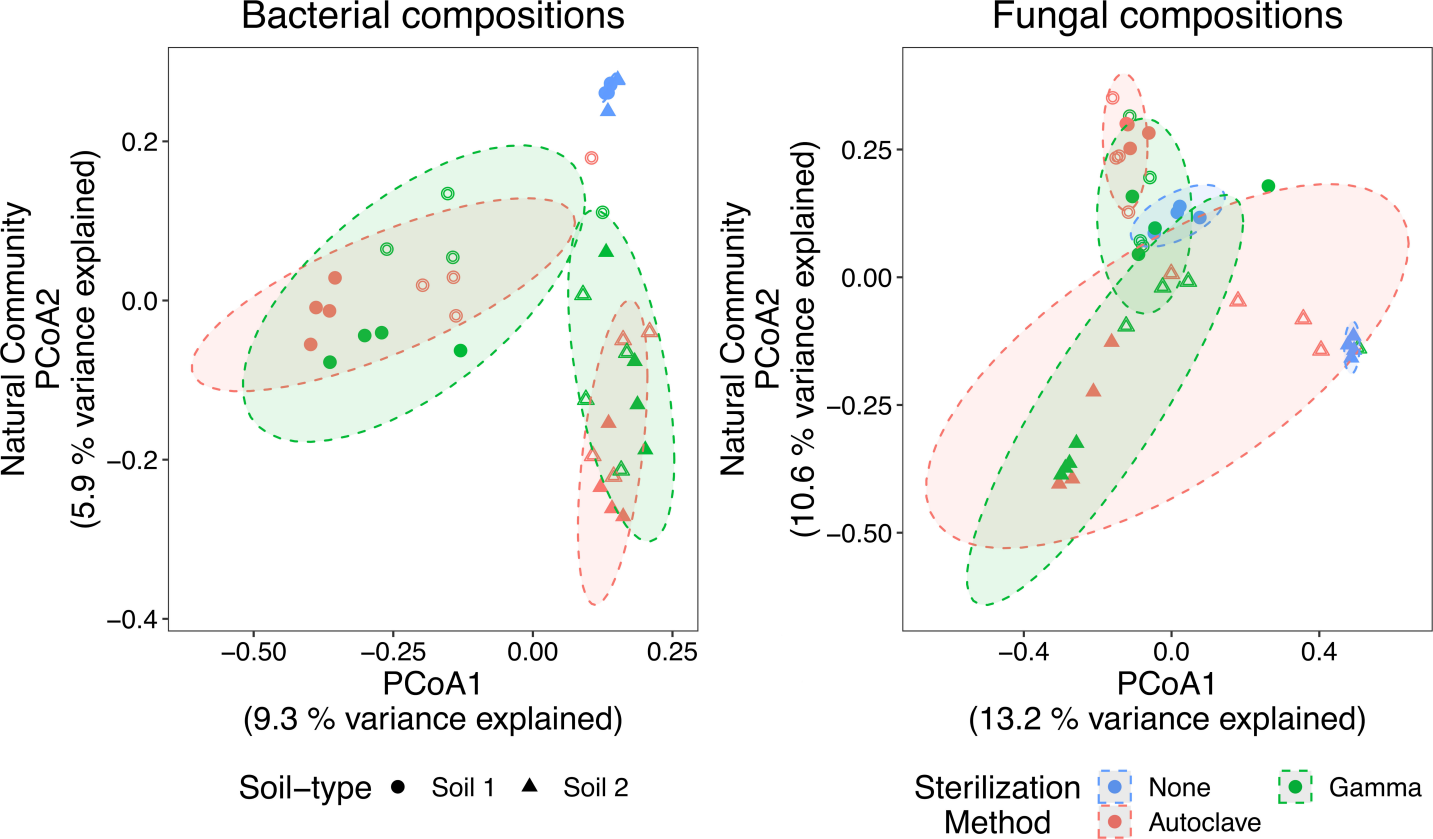
PCoA of bacterial and fungal composition following recolonization from a soil source. Points are colored according to sterilization method and the shapes refer to different soil types. Solid and hollow points refer to the no soil wash and soil wash treatments, respectively. Ninety percent ellipses are shown and are grouped according to sterilization method per soil type. Ordinations per soil are shown in Fig. S1.

We assessed the impact of both sterilization method and soil washing (i.e., autoclaving or gamma irradiation) on microbial composition following natural recolonization (Fig. 2; Fig. S1). For bacterial composition, we observed a significant effect of soil type (*F*_1,30_ = 4, *R*^2^ = 0.1, *P* ≤ 0.001), wash treatment (*F*_1,30_ = 2, *R*^2^ = 0.05, *P* ≤ 0.001), and their interaction (*F*_*1,30*_ = 2, *R*^2^ = 0.05, *P* = 0.004), but observed no significant effect of sterilization method (*P* > 0.05; interaction with soil type *P* > 0.05; Table 1). For fungal composition, we generally saw a similar pattern with a significant effect of soil type (*F*_1,30_ = 4, *R*^2^ = 0.11, *P* ≤ 0.001), wash treatment (*F*_1,30_ = 3, *R*^2^ = 0.08, *P* ≤ 0.001), and their interaction (*F*_1,30_ = 4, *R*^2^ = 0.09, *P* ≤ 0.001) and no significant effect of sterilization method. We did observe a marginally significant interaction between soil type and sterilization method (*F*_1,30_ = 2, *R*^2^ = 0.04, *P* = 0.05), and a three-way interaction between soil type, sterilization method, and wash treatment (*F*_1,30_ = 2, *R*^2^ = 0.04, *P* = 0.05), but they only explained a minor part of the composition variance (*R*^2^ = 0.04). These data suggest that sterilization method does not have a sizable impact on soil recolonization when using soil as an inoculum source, while wash treatment plays a relatively larger role in influencing microbial recolonization. Soil washing is a common practice with numerous studies using soil washing to remove inhibitory compounds (25–30). In particular, soil washing can remove various soil toxins that inhibit bacterial growth (26) and has been shown to effectively remove fungal spores (31, 32). However, microbial colonization is generally not compared between washed and unwashed soils. Regardless, our data highlight that soil washing can impact soil microbial colonization and consideration should be applied when interpreting colonization patterns in washed soil and when extrapolating to field settings.

**TABLE 1:** Statistical analysis of beta diversity for bacterial and fungal composition after natural recolonization^*a*^^,^^*b*^

Gene	Term	*F*-value	*R* ^2^	Significance
16S rRNA gene	Soil	*F*_1,30_ = 4	0.1	***
	Sterilization method	*F*_1,30_ = 1	0.03	NS
	Wash treatment	*F*_1,30_ = 2	0.05	***
	Soil * sterilization method	*F*_1,30_ = 1	0.03	NS
	Soil * wash treatment	*F*_1,30_ = 2	0.05	**
	Sterilization method * wash treatment	*F*_1,30_ = 1	0.03	NS
	Soil * sterilization method * wash treatment	*F*_1,30_ = 1	0.04	NS
ITS region	Soil	*F*_1,30_ = 4	0.11	***
	Sterilization method	*F*_1,30_ = 1	0.04	*
	Wash treatment	*F*_1,30_ = 3	0.08	***
	Soil * sterilization method	*F*_1,30_ = 2	0.04	*
	Soil * wash treatment	*F*_1,30_ = 4	0.09	***
	Sterilization method * wash treatment	*F*_1,30_ = 2	0.04	*
	Soil * sterilization method * wash treatment	*F*_1,30_ = 2	0.04	*

^*a*^ *** is ≤0.001, ** is <0.01, * is <0.05.

^*b*^ The Adonis formula for both comparisons was soil type * sterilization method * wash treatment. Comparison does not include the unsterilized soil.

To explore whether sterilization method and soil washing had a significant impact on microbial recolonization for particular taxa, we performed a SIMPER analysis on data summarized at the phylum and class level for bacteria and fungi, respectively. For bacteria, we observed a greater relative abundance of the Verrucomicrobia and Chloroflexi phyla in the gamma-irradiated recolonized soil (Table 2). For fungi, an unidentified fungal class and the Agaricomycetes were significantly higher in the gamma-irradiated soils. Wash treatment had a larger impact on the recolonization of individual taxa relative to sterilization method, particularly for fungi (Table 2).

**TABLE 2:** SIMPER analysis of taxa between sterilization methods and wash treatments^*a*^

Taxa	Autoclaved soil relative abundance (%)	Gamma-irradiated soil relative abundance (%)	*P*-value
Verrucomicrobia	1.3	2.9	0.005
Chloroflexi	0.2	0.8	0.02
Unidentified fungi	16.8	41.4	0.003
Agaricomycetes	0.01	1.3	0.001

Taxa	Not washed soil relative abundance (%)	Washed soil relative abundance (%)	*P*-value
Proteobacteria	65.8	77.0	0.012
Firmicutes	15.4	4.2	0.002
Unidentified fungi	16.0	42.4	0.003
Unclassified fungi	14.5	41.5	0.004
Sordariomycetes	29.4	4.1	0.03
Pezizomycetes	8.8	0	0.001
Leotiomycetes	3.3	0.03	0.003
Rhizophydiomycetes	0	2.3	0.001
Unidentified Chytridiomycota	0.03	2.2	0.02

^*a*^ Used data were summarized at the phylum and class levels for bacteria and fungi, respectively.

**Fig 3:**
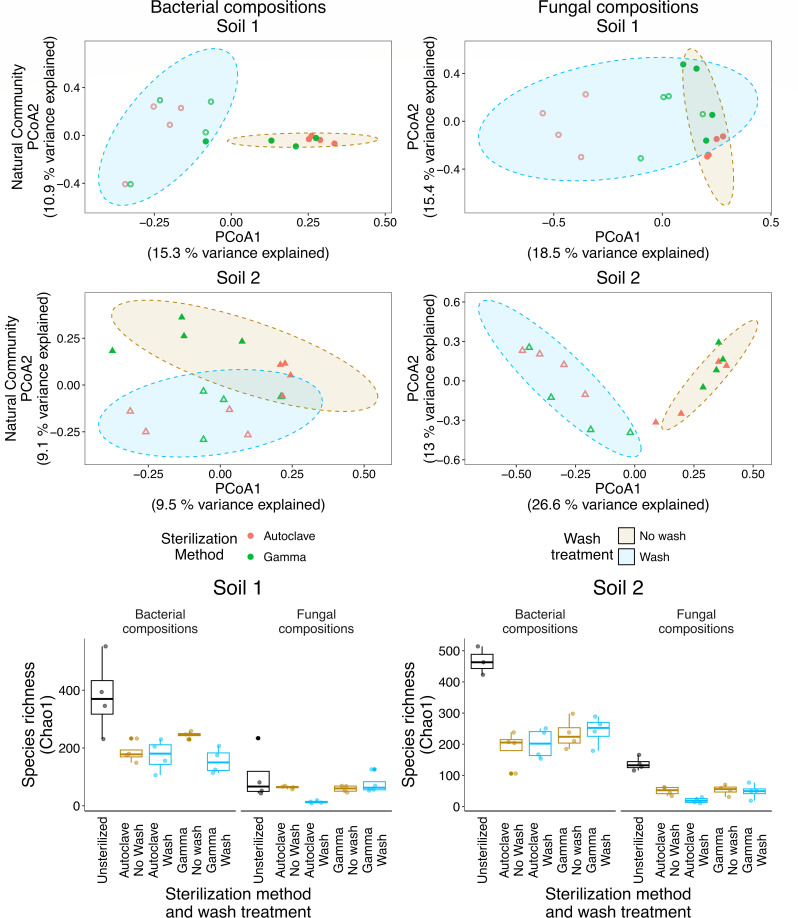
PCoA of bacterial and fungal composition clustered according to the wash treatment, and a boxplot of species richness (Chao1) for sterilization method and wash treatment. For the PCoA: the points are colored according to sterilization method, shapes represent different soil type, the fill color separates the wash treatment, and the solid and hollow points additionally refer to the no soil wash and soil wash treatments, respectively. Ninety percent ellipses are shown and are grouped according to the wash treatments.

Because of the strong influence of soil type on microbial recolonization and to further explore the impact of soil washing on microbial recolonization, we examined the impact of washing on each individual soil (Fig. 3). For each soil and for both bacterial and fungal composition, the wash treatment explained more of the compositional variance relative to the sterilization method used (Table S4). We did not detect any differences for species richness (Chao1) between soil washing treatments for the recolonized soils, but there appeared to be a trend for lower species richness in recolonized washed soils. When compared to the unsterilized soils, we saw significant differences with the recolonized washed soils for Soil 1 (bacteria: autoclave q = 0.05, gamma q = 0.03) and Soil 2 (bacteria: autoclave q = 0.04; fungi: autoclave q = 0.002). To determine whether washed or unwashed soils more closely resemble the unsterilized soil, we extracted and compared Bray-Curtis dissimilarity values. For bacteria in each soil, and fungi in Soil 1, we observed no significant differences between the wash treatments. These data agree with our previous observations that the pool of recolonizing microorganisms is usually quite distinct from the bulk soil (14, 50). However, for Soil 2 and for fungi, we did observe a significant difference (H = 18, *P* ≤ 0.001) which was primarily driven by a greater similarity between the unsterilized soil and the washed soils for each sterilization method (average Bray-Curtis dissimilarity: autoclave wash = 0.81; autoclave no wash = 0.98, gamma wash = 0.87; gamma no wash = 0.99; q-values: autoclave wash vs no wash = 0.002, gamma wash vs no wash = 0.02).

### Microbial recolonization with both sterilization methods are distinct to soil regrowth

**Fig 4:**
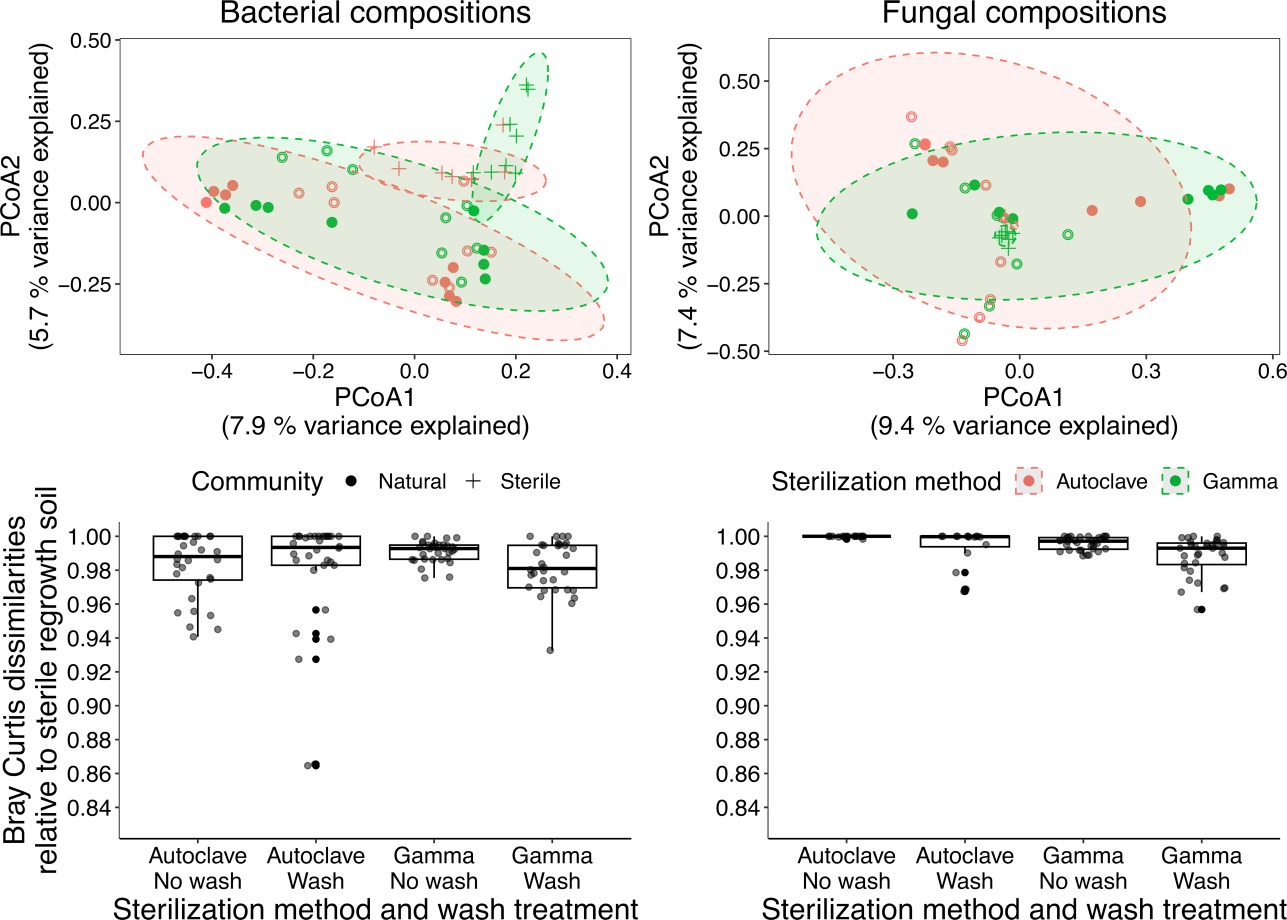
PCoA of bacterial and fungal composition for recolonized soils and sterile soil regrowth, and a boxplot of extracted Bray-Curtis dissimilarity values. For the ordination, points are colored according to sterilization method and the shapes refer to recolonized soil or sterile soil. Solid and hollow points refer to the no soil wash and soil wash treatments, respectively. Ninety percent ellipses are shown. For the boxplot, Bray-Curtis dissimilarity values relative to the sterile regrowth soil are shown.

Due to observing far fewer taxa in regrowth controls, especially following autoclaving, we sought to identify whether soil regrowth had a discernable impact on microbial composition in recolonized soils (Fig. 4). In all instances, microbial recolonization was distinct from the soil regrowth (Table S5). To identify whether recolonization in autoclaved or gamma-irradiated samples was more similar to microbial composition in regrowth controls, we extracted Bray-Curtis dissimilarity values relative to sterile soil regrowth. Generally, both bacteria and fungi in both soils were very distinct from sterile soil regrowth controls (average Bray-Curtis dissimilarity: bacteria = 0.98; fungi = 0.99) and we did not detect differences between sterilization methods and wash treatments for bacteria. For fungi, we detected significantly lower Bray-Curtis dissimilarity values for gamma-irradiated and washed soils (Table S6). However, and while significant, the actual Bray-Curtis dissimilarity values were quite similar (autoclaved and no wash = 0.9998; gamma irradiated and no wash soil = 0.9958; autoclaved and wash = 0.9937; gamma irradiated and wash = 0.9881), suggesting that the impact of soil regrowth on microbial recolonization with either sterilization technique or wash treatment is minimal.

**Fig 5:**
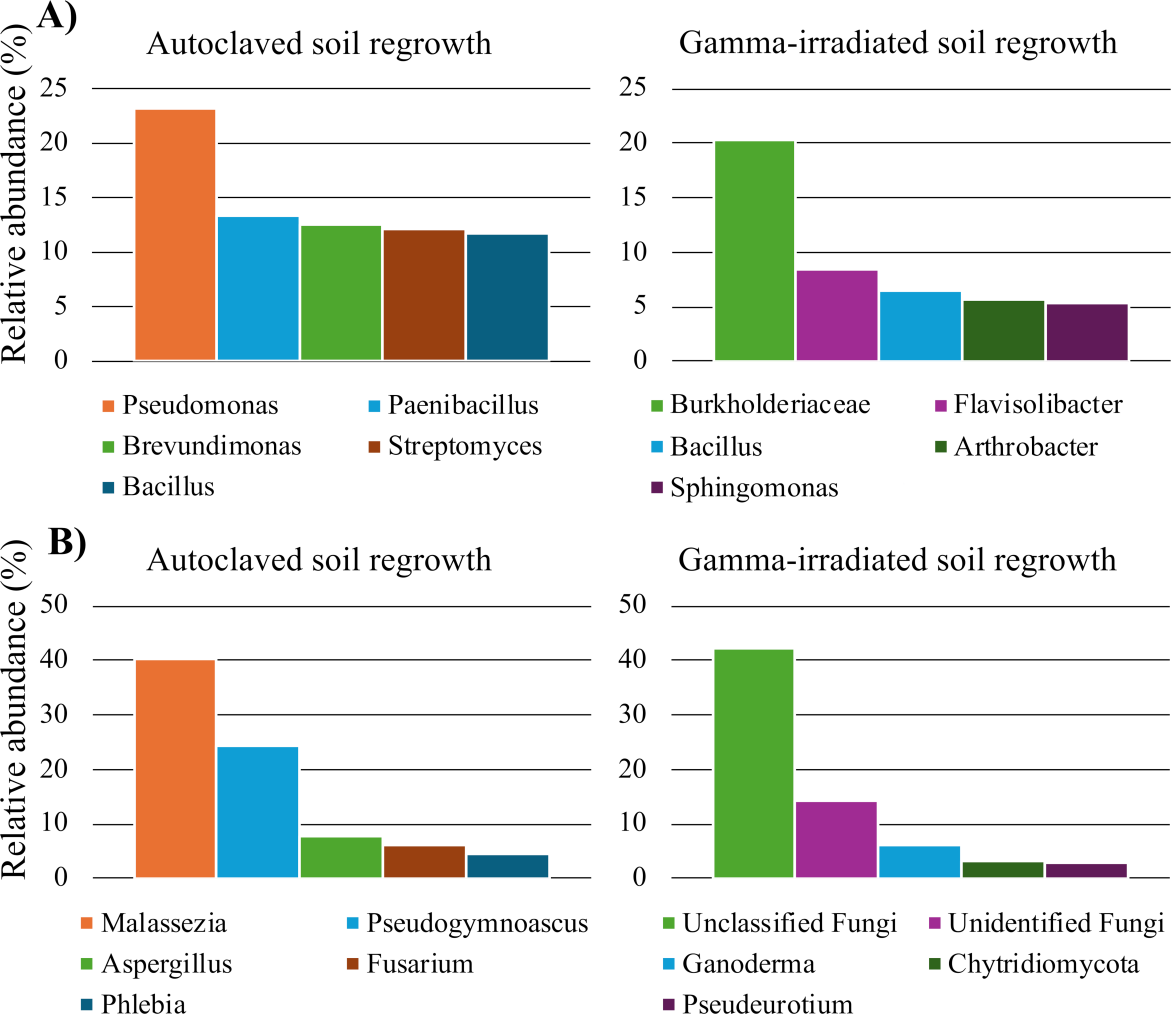
Average relative abundance of abundant bacterial (**A**) and fungal (**B**) genera in sterile regrowth soils. The top five most abundant taxa for each sterilization technique are shown.

To identify taxa that were common in our sterile regrowth, we examined the most abundant taxa in the autoclaved and gamma-irradiated sterile soils (Fig. 5). Each sterilization technique permitted the regrowth of different taxa. While gamma irradiation permitted more species richness of sterile regrowth (Fig. 1), autoclaved sterile regrowth appeared to have fewer, but more dominant, taxa (Fig. 5; Table S7). For example, bacteria assigned to the *Bacillus* genus made up 93% relative abundance in one autoclaved regrowth sample but was 1.5% in the next highest replicate (Table S7). Gamma-irradiated soil regrowth appeared to be more consistent across replicates (Table S7).

Future work will need to assess how soil characteristics and treatment conditions impact the extent of microbial regrowth in post-autoclaved soils. We have anecdotally observed differences in the degree of regrowth across the soils we have worked with. We never observe detectable DNA in soils directly post-autoclaving and, in some cases, DNA stays below detection after many weeks of incubation. As a limited-scope follow-on study to this work, we assessed the amount of detectable DNA via Qubit following incubation of microcosms in the absence of any microbial reinoculation (Fig. S2; Table S8). Relative to DNA concentrations in initial soils, regrowth DNA was always very low, but varied across soil conditions and treatment conditions. For instance, it appeared that lower soil thickness at autoclaving and more autoclave cycles (three vs one) led to less regrowth after 7 weeks. Future work that more robustly assesses the conditions affecting regrowth will improve our ability to generate diverse and effective model soil systems.

### Conclusions

Biotically cleared soils have been used for decades as a medium to study microbial colonization and persistence and relationships between biodiversity and community function (10, 11, 51–53). Biotically cleared soils act as soil-like environments that more closely mimic natural conditions compared to culturing using commercially available media. Sterilization methods are still hotly debated, with gamma irradiation often touted as the gold standard. In this study, we have shown that (i) gamma-irradiated soils permitted greater microbial regrowth following sterilization, (ii) sterilization method had minimal impact on natural microbial recolonization, (iii) soil washing had a greater impact on microbial colonization relative to sterilization method, and (iv) fungi were particularly sensitive to autoclaving when examining our soil regrowth controls. The greater efficacy of autoclaving could be due to the repeated autoclaving steps which permit microbial regrowth and sporulation before autoclaving again (17). These data suggest that autoclaving and gamma irradiation are both effective techniques for biotically clearing soil, and that autoclaving performed marginally better when considering soil regrowth.

## SUPPLEMENTAL MATERIAL

Supplemental Information msphere.00476-24-s0001.docx Figures S1 and S2 and Tables S1 to S8.

ASM does not own the copyrights to Supplemental Material that may be linked to, or accessed through, an article. The authors have granted ASM a non-exclusive, world-wide license to publish the Supplemental Material files. Please contact the corresponding author directly for reuse.
