# Supplementary material for: Autoclaving is at least as effective as gamma irradiation for biotic clearing and intentional microbial recolonization of soil
Source: mSphere. 2024 Jul 9;9(7):e00476-24. doi: 10.1128/msphere.00476-24 (PMC11288020; doi:10.1128/msphere.00476-24)
Supplement: Supplemental Information — Figures S1 and S2 and Tables S1 to S8. [file msphere.00476-24-s0001.docx]

Autoclaving is at least as effective as gamma irradiation for biotic clearing and intentional microbial recolonization of soil

William L King^1,2,3^, Emily M. Grandinette^1^, Olivia Trase^4,5^, M. Laura Rolon^6^, Howard M. Salis^7^, Harlow Wood^8^, Terrence H. Bell^1,8*^

^1^Department of Plant Pathology and Environmental Microbiology, The Pennsylvania State University, University Park, PA, USA

^2^School of Integrative Plant Science, Cornell University, Ithaca, NY 14853, USA

^3^School of Biological Sciences, University of Southampton, Southampton, SO17 1BJ, UK

^4^Department of Entomology, The Pennsylvania State University, University Park, PA, 16802, USA

^5^Intercollege Graduate Degree Program in Ecology, The Pennsylvania State University, University Park, PA, 16802, USA

^6^Department of Food Science, The Pennsylvania State University, University Park, PA, 16802, USA

^7^Department of Agricultural and Biological Engineering, The Pennsylvania State University, University Park, PA, USA

^8^Department of Physical & Environmental Sciences, University of Toronto Scarborough, Toronto, ON, M1C1A4, Canada

*Correspondance: terrence.bell@utoronto.ca

Supplementary Table 1: Soil physiochemical data

| Soil | pH | N (%) | C (%) | P | K | Mg | Ca | OM (%) | Zn | Cu | S | Textural class |
| --- | --- | --- | --- | --- | --- | --- | --- | --- | --- | --- | --- | --- |
| Soil 1  (Forest soil) | 5.1 | 0.389 | 4.412 | 17 | 81 | 117 | 901.3 | 6.76 | 1.8 | 1 | 8.7 | Loam |
| Soil 2  (Farm soil) | 6.4 | 0.146 | 1.322 | 21 | 106 | 178 | 1430.9 | 2.48 | 1.4 | 2.2 | 6.9 | Silty clay |
| Autoclaved  soil 1 | 7.4 | 0.218 | 6.782 | 17 | 61 | 141 | 10711.7 | 3.87 | 1.3 | 0.8 | 67.2 | NA |
| Autoclaved  soil 2 | 7.9 | 0.099 | 4.223 | 18 | 82 | 135 | 7373.6 | 1.56 | 1.2 | 1.1 | 49.8 | NA |
| Irradiated  soil 1 | 7.6 | 0.261 | 6.546 | 20 | 80 | 178 | 8401.3 | 4.17 | 1.4 | 0.9 | 53.9 | NA |
| Irradiated  soil 2 | 8 | 0.108 | 3.677 | 20 | 93 | 194 | 5716.2 | 1.61 | 1.4 | 2 | 49.7 | NA |

Supplementary Table 2: Bacterial alpha diversity comparisons. Comparisons per performed for each Soil. The Natural recolonization treatment and Sterile regrowth treatment were compared individually. The ANOVA formula was Sterilization * Wash and followed by a Tukey HSD post hoc. NS is not significant.

| Soil | Metric | Natural recolonization ANOVA | Sterile regrowth Kruskal-Wallis test |
| --- | --- | --- | --- |
| Soil 1 | Richness | Sterilization: NS  Wash: F_1,11_ = 6, p = 0.03  Sterilization*Wash: NS, p = 0.07 | H = 4, p = 0.04 |
|  | Diversity | Sterilization: NS  Wash: NS  Sterilization*Wash: NS | NS |
| Soil 2 | Richness | Sterilization: NS  Wash: NS  Sterilization*Wash: NS | H = 5, p = 0.02 |
|  | Diversity | Sterilization: NS^  Wash: NS^ | H = 5, p = 0.02 |

^Kruskal-Wallis test

Supplementary Table 3: Fungal alpha diversity comparisons. Comparisons per performed for each Soil. The Natural recolonization treatment and Sterile regrowth treatment were compared individually. The ANOVA formula was Sterilization * Wash and followed by a Tukey HSD post hoc. NS is not significant.

| Soil | Metric | Natural recolonization ANOVA | Tukey HSD post hoc comparison | Sterile regrowth Kruskal-Wallis |
| --- | --- | --- | --- | --- |
| Soil 1 | Richness | Sterilization: F_1,11_ = 34, p ≤ 0.001  Wash: F_1,11_ = 26, p ≤ 0.001  Sterilization*Wash: F_1,11_ = 43, p ≤ 0.001 | Autoclave No Wash vs. Gamma No Wash: NS  Autoclave Wash vs. Autoclave No Wash: q ≤ 0.001  Gamma Wash vs. Autoclave No Wash: NS  Autoclave Wash vs. Gamma No Wash: q ≤ 0.001  Gamma Wash vs. Gamma No Wash: NS  Autoclave Wash vs. Gamma Wash: q ≤ 0.001 | NS^ |
|  | Diversity | Sterilization: F_1,11_ = 10, p = 0.008  Wash: F_1,11_ = 37, p ≤ 0.001  Sterilization*Wash: F_1,11_ = 43, p ≤ 0.001 | Autoclave No Wash vs. Gamma No Wash: NS  Autoclave Wash vs. Autoclave No Wash: q ≤ 0.001  Gamma Wash vs. Autoclave No Wash: NS  Autoclave Wash vs. Gamma No Wash: q ≤ 0.001  Gamma Wash vs. Gamma No Wash: NS  Autoclave Wash vs. Gamma Wash: q ≤ 0.001 | NS^ |
| Soil 2 | Richness | Sterilization: NS, p = 0.08  Wash: NS, p = 0.06  Sterilization*Wash: NS | NA | NS^ |
|  | Diversity | Sterilization: NS, p = 0.0503  Wash: NS  Sterilization*Wash: NS | NA | NS^ |

^low replicate numbers in the autoclaved sterile regrowth soils

Supplementary Figure 1: Principal Coordinate Analysis (PCoA) ordinations of bacterial and fungal compositions.

Supplementary Table 4: Beta diversity comparisons between Wash treatments in each soil for the natural recolonization treatment. NS is not significant. Adonis formula was Wash * Sterilization

| Gene | Soil | Term | Statistic |
| --- | --- | --- | --- |
| 16S rRNA | Soil 1 | Wash  Sterilization  Wash*Sterilization | F_1,14_ = 2, R^2^ = 0.14, p ≤ 0.001  NS  NS |
|  | Soil 2 | Wash  Sterilization  Wash*Sterilization | F_1,14_ = 1, R^2^ = 0.09, p ≤ 0.001  F_1,14_ = 1, R^2^ = 0.08, p = 0.004  F_1,14_ = 1, R^2^ = 0.08, p ≤ 0.001 |
| ITS region | Soil 1 | Wash  Sterilization  Wash*Sterilization | F_1,14_ = 3, R^2^ = 0.14, p ≤ 0.001  F_1,14_ = 2, R^2^ = 0.09, p = 0.03  F_1,14_ = 2, R^2^ = 0.11, p = 0.009 |
|  | Soil 2 | Wash  Sterilization  Wash*Sterilization | F_1,14_ = 5, R^2^ = 0.24, p ≤ 0.001  NS  NS |

Supplementary Table 5: Beta diversity comparisons between sterilized soil regrowth and the natural recolonization treatment. NS is not significant. Adonis formula was Community *Sterilization*Wash

| Gene | Soil | PERMANOVA |
| --- | --- | --- |
| 16S rRNA | Soil 1 | Community: F_1,23_ = 2, R^2^ = 0.09, p ≤ 0.001  Sterilization: NS  Wash: F_1,23_ = 2, R^2^ = 0.08, p ≤ 0.001  Community*Sterilization: NS, p = 0.08  Sterilization*Wash: NS |
|  | Soil 2 | Community: F_1,23_ = 2, R^2^ = 0.09, p ≤ 0.001  Sterilization: F_1,23_ = 1, R^2^ = 0.05, p = 0.01  Wash: F_1,23_ = 1, R^2^ = 0.05, p = 0.01  Community*Sterilization: F_1,23_ = 1, R^2^ = 0.05, p = 0.04  Sterilization*Wash: NS; p = 0.054 |
| ITS region | Soil 1 | Community: F_1,22_ = 2, R^2^ = 0.09, p ≤ 0.001  Sterilization: F_1,22_ = 2, R^2^ = 0.06, p = 0.008  Wash: F_1,22_ = 2, R^2^ = 0.09, p ≤ 0.001  Community*Sterilization: F_1,22_ = 2, R^2^ = 0.07, p = 0.002  Sterilization*Wash: F_1,22_ = 2, R^2^ = 0.07, p = 0.003 |
|  | Soil 2 | Community: F_1,22_ = 3, R^2^ = 0.11, p ≤ 0.001  Sterilization: NS  Wash: F_1,22_ = 4, R^2^ = 0.17, p ≤ 0.001  Community*Sterilization: F_1,22_ = 2, R^2^ = 0.06, p = 0.02  Sterilization*Wash: NS |

Supplementary Table 6: Bray-Curtis dissimilarity comparisons between Sterilization methods and wash treatment. NS is not significant and NA is not applicable as the overall test was not significant.

| Gene | Overall comparison | Dunn post hoc comparison |
| --- | --- | --- |
| 16S rRNA | NS | NA |
| ITS region | d.f. = 3, H = 34, p ≤ 0.001 | Autoclave No Washed vs Autoclave Washed: Z = 2.1, q = 0.04  Autoclave No Washed vs Gamma No Washed: Z = 3.7, q ≤ 0.001  Autoclave No Washed vs Gamma Washed: Z = 5.7, q ≤ 0.001  Autoclave Washed vs Gamma No Washed: NS  Autoclave Washed vs Gamma Washed: Z = 3.3, q = 0.002  Gamma No Washed vs Gamma Washed: Z = 2.5, q = 0.02 |

Supplementary Table 7: Top five most abundant genera in sterile soil regrowth samples. The top five for both autoclaved and gamma-irradiated soils are shown. Gamma-irradiated replicates are shaded grey.

| Taxa | Replicate relative abundance (%) | | | | | | | | | | | | |
| --- | --- | --- | --- | --- | --- | --- | --- | --- | --- | --- | --- | --- | --- |
| Pseudomonas | 0.72 | 98.19 | 45.17 | | 0.00 | | 41.36 | | 0.00 | | 0.00 | | 0.30 |
| Paenibacillus | 0.00 | 0.00 | 3.75 | | 0.00 | | 3.21 | | 99.69 | | 0.00 | | 0.00 |
| Brevundimonas | 0.00 | 0.00 | 0.00 | | 0.00 | | 0.00 | | 0.00 | | 98.62 | | 1.71 |
| Streptomyces | 0.00 | 0.00 | 0.00 | | 0.00 | | 0.00 | | 0.00 | | 0.49 | | 96.51 |
| Bacillus | 92.55 | 0.00 | 0.00 | | 1.48 | | 0.00 | | 0.00 | | 0.00 | | 0.03 |
| Burkholderiaceae | 7.76 | 1.26 | 0.33 | | 0.00 | | 6.84 | | 60.28 | | 12.33 | | 74.08 |
| Flavisolibacter | 23.05 | 0.21 | 0.00 | | 0.12 | | 44.45 | | 0.00 | | 0.00 | | 0.00 |
| Bacillus | 11.74 | 1.94 | 0.03 | | 4.41 | | 15.89 | | 2.63 | | 11.92 | | 3.68 |
| Arthrobacter | 0.00 | 0.00 | 44.82 | | 0.00 | | 0.00 | | 0.00 | | 0.00 | | 0.00 |
| Sphingomonas | 0.14 | 13.71 | 9.60 | | 8.46 | | 0.00 | | 8.95 | | 0.87 | | 0.80 |
| Taxa | Replicate | | | | | | | | | | | | |
| Malassezia | 57.41 | | 1.40 | | | | 2.35 | | | | 100.00 | | |
| Pseudogymnoascus | 0.00 | | 97.61 | | | | 0.00 | | | | 0.00 | | |
| Aspergillus | 18.07 | | 0.99 | | | | 12.22 | | | | 0.00 | | |
| Fusarium | 0.00 | | 0.00 | | | | 24.94 | | | | 0.00 | | |
| Phlebia | 17.86 | | 0.00 | | | | 0.00 | | | | 0.00 | | |
| Unclassified Fungi | 36.30 | 42.10 | 49.30 | 32.26 | | 43.33 | | 62.55 | | 39.96 | | 32.14 | |
| Unidentified Fungi | 11.73 | 16.87 | 18.77 | 28.60 | | 6.34 | | 7.37 | | 9.63 | | 15.51 | |
| Ganoderma | 8.52 | 4.77 | 0.00 | 0.00 | | 5.27 | | 3.83 | | 16.46 | | 9.42 | |
| Unidentified Chytridiomycota | 0.04 | 3.46 | 1.69 | 8.81 | | 9.79 | | 0.00 | | 0.82 | | 1.28 | |
| Pseudeurotium | 0.41 | 2.10 | 6.30 | 13.09 | | 0.00 | | 0.00 | | 0.00 | | 0.00 | |

Supplementary Figure 2: Amount of DNA detectable via Qubit after extraction from soils. A) DNA quantified from soils post-collection (n=7), directly post-autoclaving (n=7), and after 7 weeks incubation in closed microcosms with no inoculation. B) DNA quantified across the seven collected soil types after 7 weeks incubation in closed microcosms with no inoculation (average of n=2 for each). C) DNA quantified across different treatment conditions applied to soil-type TS-7. Standard conditions included field-level moisture, 3 autoclave cycles, and 1.5” soil thickness at autoclaving. Other treatments each involve a single modification from standard conditions (average of n=2 for each).

Supplementary Table 8: Traits for soils used in Supplementary Figure 2. Characteristics are shown both before and after autoclaving. All traits assessed at A&L Canada Laboratories Inc. (London, ON, Canada) following standard protocols.

|  | Relative to autoclaving | Organic matter (%) | P (ppm; Bray-P1) | Al (ppm) | K (ppm) | Mg (ppm) | Ca (ppm) | Na (ppm) | pH | CEC |
| --- | --- | --- | --- | --- | --- | --- | --- | --- | --- | --- |
| TS-1 | Pre | 3.6 | 11 | 324 | 49 | 94 | 3560 | 14 | 7.6 | 18.8 |
|  | Post | 3.5 | 31 | 322 | 46 | 56 | 3590 | 18 | 7.3 | 18.6 |
| TS-2 | Pre | 5.9 | 19 | 239 | 69 | 118 | 3880 | 14 | 7.4 | 20.6 |
|  | Post | 5.9 | 47 | 213 | 90 | 77 | 3490 | 17 | 7.2 | 19.3 |
| TS-3 | Pre | 3.2 | 51 | 810 | 30 | 80 | 1040 | 15 | 5.4 | 8.4 |
|  | Post | 3 | 92 | 846 | 43 | 93 | 1320 | 21 | 6 | 8.8 |
| TS-4 | Pre | 3.6 | 6 | 328 | 50 | 72 | 3810 | 13 | 7.8 | 19.8 |
|  | Post | 3.3 | 17 | 363 | 52 | 48 | 4060 | 18 | 8 | 20.9 |
| TS-5 | Pre | 2.9 | 4 | 42 | 31 | 152 | 7200 | 33 | 7.7 | 37.5 |
|  | Post | 2.3 | 12 | 38 | 29 | 110 | 7180 | 33 | 8 | 37 |
| TS-6 | Pre | 5.5 | 25 | 739 | 46 | 122 | 2270 | 25 | 6 | 13.8 |
|  | Post | 5.2 | 43 | 493 | 51 | 91 | 1570 | 22 | 5.6 | 10 |
| TS-7 | Pre | 7.9 | 8 | 51 | 43 | 151 | 7200 | 21 | 7.6 | 37.4 |
|  | Post | 8 | 14 | 37 | 54 | 106 | 7090 | 24 | 7.8 | 36.6 |
